# Supplementary material for: Using AI to measure Parkinson’s disease severity at home
Source: NPJ Digit Med. 2023 Aug 23;6:156. doi: 10.1038/s41746-023-00905-9 (PMC10444879; doi:10.1038/s41746-023-00905-9)
Supplement: Supplementary file 1 — Supplementary Information [file 41746_2023_905_MOESM1_ESM.pdf]

# Using AI to Measure Parkinson's Disease Severity at Home

Md Saiful Islam[1,3*], Wasifur Rahman[1], Abdelrahman Abdelkader[1], Sangwu Lee[1], Phillip T. Yang[2], Jennifer Lynn Purks[2], Jamie Lynn Adams[2], Ruth B. Schneider[2], Earl Ray Dorsey[2], Ehsan Hoque[1]

[1*]Department of Computer Science, University of Rochester, 250 Hutchinson Road, Rochester, 14620, New York, United States.
[2]Department of Neurology, University of Rochester Medical Center, 601 Elmwood Avenue, Rochester, 14642, New York, United States.
[3]Department of Computer Science and Engineering, Bangladesh University of Engineering & Technology, West Palashi, Dhaka, 1000, Bangladesh.

*Corresponding author(s). E-mail(s): mislam6@ur.rochester.edu;

## Supplementary Note 1

We used Python to conduct the experiments. The versions for each package used are listed below:

- click=8.1.3
- cuda-cudart=11.8.89
- cuda-cupti=11.8.87
- cuda-libraries=11.8.0
- cuda-nvrtc=11.8.89
- cuda-nvtx=11.8.86
- cuda-runtime=11.8.0
- imbalanced-learn=0.10.1
- lightgbm=3.3.5
- matplotlib=3.7.1
- mediapipe=0.8.10
- numba=0.57.0

- numpy=1.24.3
- pandas=2.0.2
- pip=22.3.1
- python=3.9.13
- pytorch=2.0.1
- pytorch-cuda=11.8
- scikit-learn=1.2.2
- scipy=1.10.1
- seaborn=0.12.2
- shap-hypetune=0.2.6
- shap=0.41.0
- torchvision=0.15.2
- tqdm=4.65.0
- wandb=0.15.4
- xgboost=1.7.5

| **Comparison Groups** | **MAE** | **MSE** | **Accuracy** | **Kendall's $\tau$** | **MAPE** | **PCC** | **Spearman's $\rho$** |
|---|---|---|---|---|---|---|---|
| Expert vs Ground Truth | 0.265 | 0.286 | 74.5% | 0.816 | 12.79% | 0.863 | 0.862 |
| Expert vs Expert | **0.531** | 0.623 | **51.35%** | **0.65** | **25.78%** | **0.722** | **0.717** |
| Non-Expert vs Expert | 0.873 | 1.33 | 33.42% | 0.502 | 46.2% | 0.571 | 0.564 |
| Non-Expert vs Ground Truth | 0.826 | 1.233 | 36.03% | 0.534 | 44.06% | 0.609 | 0.598 |
| Best Model vs Ground Truth | 0.580 | **0.536** | 50.92% | 0.515 | 32.01% | 0.656 | 0.643 |

Supplementary Table 1: **Performance of experts, non-experts, and the model.** "Expert vs Ground Truth" assesses the average agreement between an expert's ratings and the ground-truth severity scores. "Expert vs Expert" measures the consistency of ratings among pairs of experts. "Non-Expert vs Expert" examines the association between ratings from non-experts and experts on average. "Non-Expert vs Ground Truth" evaluates the performance of non-experts by comparing their ratings to the ground-truth scores. Lastly, "Best Model vs Ground Truth" measures the performance of the best model against the ground-truth scores. Considering that the ground-truth scores are derived from the experts' ratings, it is expected that "Expert vs Ground Truth" would exhibit the strongest association. Among the other comparison groups, the best metrics are highlighted as **bold**, while the second best metrics are underlined. Some of the metrics are abbreviated for the simplicity of presentation. MAE: Mean Absolute Error (points), MSE: Mean Squared Error (points), MAPE: Mean Absolute Percentage Error (%), PCC: Pearson's Correlation Coefficient.

| Subgroup | Attribute | Severity Score | | | | | Total |
|---|---|---|---|---|---|---|---|
| | | 0 | 1 | 2 | 3 | 4 | |
| | Number of videos, n (%) | 108 (22.1%) | 181 (37.0%) | 141 (28.8%) | 54 (11.0%) | 5 (1.0%) | 489 (100%) |
| Sex, n (%) | Male | 48 (44.4%) | 100 (55.2%) | 80 (56.7%) | 35 (64.8%) | 4 (80%) | 267 (54.6%) |
| | Female | 60 (55.6%) | 81 (44.8%) | 61 (43.3%) | 19 (35.2%) | 1 (20%) | 222 (45.4%) |
| Ethnicity, n (%) | white | 91 (84.3%) | 169 (93.4%) | 136 (96.5%) | 51 (94.4%) | 5 (100%) | 452 (92.4%) |
| | non-white | 17 (15.7%) | 12 (6.6%) | 5 (3.5%) | 3 (5.6%) | 0 (0%) | 37 (7.6%) |
| Recording environment, n (%) | Home | 87 (80.6%) | 136 (75.1%) | 119 (84.4%) | 46 (85.2%) | 5 (100%) | 393 (80.4%) |
| | Clinic | 21 (19.4%) | 45 (24.9%) | 22 (15.6%) | 8 (14.8%) | 0 (0%) | 96 (19.6%) |

Supplementary Table 2: **Distribution of ground truth severity scores across demographic groups.** The severity score is an integer ranging from 0 (lowest severity) to 4 (highest severity).

| Feature selection method | Base model | MAE | MSE | Accuracy | Kendal's $\tau$ | MAPE | PCC | Spearman's r |
|---|---|---|---|---|---|---|---|---|
| BoostRFE | LightGBM | **0.5802** | 0.5364 | 50.92% | **0.5147** | **32.01%** | **0.6563** | **0.6429** |
| | XGBoost | 0.5855 | **0.5251** | **51.94%** | 0.5032 | 33.41% | 0.6446 | 0.6309 |
| BoostRFA | LightGBM | 0.5993 | 0.5679 | 50.92% | 0.4911 | 33.56% | 0.63 | 0.6149 |
| | XGBoost | 0.5861 | 0.5586 | **51.94%** | 0.5044 | 32.14% | 0.6388 | 0.6329 |

Supplementary Table 3: **Effect of different feature selectors.** The performance of the best model (LightGBM regressor) is reported with different feature selectors we experimented with. The best metrics are highlighted as **bold**. Some of the metrics are abbreviated for the simplicity of presentation. MAE: Mean Absolute Error (points), MSE: Mean Squared Error (points), MAPE: Mean Absolute Percentage Error (%), PCC: Pearson's Correlation Coefficient.

| Name of the hyper-parameter | Distribution | Values/Range | Best Value |
|---|---|---|---|
| learning rate | Uniform | [0.01, 0.3] | 0.01313 |
| maximum depth of decision tree | Uniform (integer) | [3, 18] | 3 |
| number of estimators | Uniform (integer) | [25, 1000] | 611 |
| random state | Uniform (integer) | [0, 8192] | 42 |
| seed | Uniform (integer) | [0, 8192] | 42 |
| use feature selection? | N/A | {"yes", "no"} | "yes" |
| method of feature selection | N/A | {"BoostRFE", "BoostRFA"} | "BoostRFE" |
| base model for feature selector | N/A | {"XGBoost", "LightGBM"} | "LightGBM" |
| number of top features | Uniform (integer) | [2, 53] | 22 |
| subsample | Uniform | [0.1, 1] | 0.8 |
| use feature scaling? | N/A | {"yes", "no"} | "yes" |
| method of feature scaling | N/A | {"StandardScaler", "MinMaxScaler"} | "StandardScaler" |
| use minority oversample (SMOTE) | N/A | {"yes", "no"} | "no" |

Supplementary Table 4: **Hyper-parameter search space for the best model (LightGBM).** For hyper-parameters with a specific set of pre-defined values, the distribution is not applicable (N/A). [a,b] represents a range of values from a to b; {.} represents a set of discrete values. Best value column indicates the value of hyperparameters used to train the best model.

| **Comparison groups** | **group 1 mean (std)** | **group 2 mean (std)** | **test-statistic** | **p-value** |
|---|---|---|---|---|
| Original vs slightly blurred videos | 0.96 (0.031) | 0.958 (0.033) | 0.289 | 0.773 |
| Original vs substantially blurred videos* | 0.96 (0.031) | 0.935 (0.08) | 2.624 | 0.009 |
| Slightly blurred vs substantially blurred videos* | 0.958 (0.033) | 0.935 (0.08) | 2.445 | 0.016 |
| Original vs videos with small added noise* | 0.96 (0.031) | 0.897 (0.124) | 4.530 | $1 \times 10^{-5}$ |
| Original vs videos with high added noise* | 0.96 (0.031) | 0.784 (0.170) | 9.364 | $5 \times 10^{-17}$ |
| Videos with small vs high added noise* | 0.897 (0.124) | 0.784 (0.170) | 4.944 | $2 \times 10^{-6}$ |

Supplementary Table 5: **Test statistics for assessing MediaPipe performance.** Details of the statistical tests for assessing the difference in MediaPipe confidence scores across video groups of different quality. We used a two-tailed paired sample t-test to report the statistical significance. The comparison groups with a star demonstrated statistically significant differences (at level of significance $\alpha = 0.05$).
