# Supplementary material for: Using AI to measure Parkinson’s disease severity at home
Source: NPJ Digit Med. 2023 Aug 23;6:156. doi: 10.1038/s41746-023-00905-9 (PMC10444879; doi:10.1038/s41746-023-00905-9)
Supplement: Supplementary file 2 — Reporting Summary [file 41746_2023_905_MOESM2_ESM.pdf]

nature portfolio

Corresponding author(s): Md Saiful Islam

Last updated by author(s): Jun 19, 2023

Reporting Summary

Nature Portfolio wishes to improve the reproducibility of the work that we publish. This form provides structure for consistency and transparency in reporting. For further information on Nature Portfolio policies, see our Editorial Policies and the Editorial Policy Checklist.

Statistics

For all statistical analyses, confirm that the following items are present in the figure legend, table legend, main text, or Methods section.

n/a	Confirmed	
☐	☒	The exact sample size (*n*) for each experimental group/condition, given as a discrete number and unit of measurement
☒	☐	A statement on whether measurements were taken from distinct samples or whether the same sample was measured repeatedly
☐	☒	The statistical test(s) used AND whether they are one- or two-sided<br>*Only common tests should be described solely by name; describe more complex techniques in the Methods section.*
☒	☐	A description of all covariates tested
☐	☒	A description of any assumptions or corrections, such as tests of normality and adjustment for multiple comparisons
☐	☒	A full description of the statistical parameters including central tendency (e.g. means) or other basic estimates (e.g. regression coefficient) AND variation (e.g. standard deviation) or associated estimates of uncertainty (e.g. confidence intervals)
☐	☒	For null hypothesis testing, the test statistic (e.g. *F*, *t*, *r*) with confidence intervals, effect sizes, degrees of freedom and *P* value noted<br>*Give P values as exact values whenever suitable.*
☒	☐	For Bayesian analysis, information on the choice of priors and Markov chain Monte Carlo settings
☒	☐	For hierarchical and complex designs, identification of the appropriate level for tests and full reporting of outcomes
☐	☒	Estimates of effect sizes (e.g. Cohen's *d*, Pearson's *r*), indicating how they were calculated

Our web collection on statistics for biologists contains articles on many of the points above.

Software and code

Policy information about availability of computer code

Data collection	We used parktest.net to collect data for this study.
Data analysis	We used Python to analyze the data. The following softwares were used:<br>click=8.1.3, imbalanced-learn=0.10.1, lightgbm=3.3.5, matplotlib=3.7.1, mediapipe=0.8.10, numpy=1.24.3, pandas=2.0.2, python=3.9.13, scikit-learn=1.2.2, scipy=1.10.1, shap-hypetune=0.2.6, shap=0.41.0, wandb=0.15.4, xgboost=1.7.5

For manuscripts utilizing custom algorithms or software that are central to the research but not yet described in published literature, software must be made available to editors and reviewers. We strongly encourage code deposition in a community repository (e.g. GitHub). See the Nature Portfolio guidelines for submitting code & software for further information.

Data

Policy information about availability of data

All manuscripts must include a data availability statement. This statement should provide the following information, where applicable:

- Accession codes, unique identifiers, or web links for publicly available datasets
- A description of any restrictions on data availability
- For clinical datasets or third party data, please ensure that the statement adheres to our policy

Unfortunately, we are unable to share the raw videos due to the Health Insurance Portability and Accountability Act (HIPAA) compliance. However, we are committed to sharing the extracted features upon receiving an email request at rochesterhci@gmail.com. The features will be provided in a structured format that

can be easily integrated with existing machine-learning workflows.

For potential collaboration, we welcome interested individuals or groups to reach out to us at mehoque@cs.rochester.edu. Depending on the specifics of the collaboration, we may be able to share some additional data beyond the extracted features.

Research involving human participants, their data, or biological material

Policy information about studies with human participants or human data. See also policy information about sex, gender (identity/presentation), and sexual orientation and race, ethnicity and racism.

Reporting on sex and gender	In this study, sex was self-reported by the participants. Out of 250 participants, 137 were male whereas 113 were female. Sex was not a targeted criterion for data collection and experiment design.<br><br>We measured whether the AI model developed in this study demonstrates bias to any particular sex group. Our model achieved a mean absolute error (MAE) of 0.60 (standard deviation, std = 0.48) for male subjects (n = 267) 0.55 (std = 0.39) for female subjects (n = 222), indicating relatively accurate predictions for both sex groups Furthermore, we conducted statistical test (i.e., two-sample two-tailed t-test) to compare the errors across the two groups and found no significant difference (p-value = 0.21).
Reporting on race, ethnicity, or other socially relevant groupings	To capture race information, we used broadly used sets of races: White, Asian, Black or African American, American Indian or Alaska Native, and Others. Race was self-reported, where the participants could choose one of the above-mentioned options.<br><br>Out of 250 participants in this study, 230 self-identified them as White, 7 as Asian, 3 as African American, 2 as American Indian or Alaska Native, 1 as other. We did not have race information for 7 participants.
Population characteristics	Majority of the study (222 out of 250) participants were elderly (i.e., 50 years or older). 172 participants had Parkinson's disease while 78 were healthy.
Recruitment	People who have enrolled in Parkinson's disease study registry of University of Rochester Medical Centre were reached out by clinical study coordinators.
Ethics oversight	University of Rochester

Note that full information on the approval of the study protocol must also be provided in the manuscript.

Field-specific reporting

Please select the one below that is the best fit for your research. If you are not sure, read the appropriate sections before making your selection.

☐ Life sciences ☒ Behavioural & social sciences ☐ Ecological, evolutionary & environmental sciences

For a reference copy of the document with all sections, see nature.com/documents/nr-reporting-summary-flat.pdf

Behavioural & social sciences study design

All studies must disclose on these points even when the disclosure is negative.

Study description	The study analyzes videos of participants to measure the motor symptom severity of Parkinson's disease. The severity rating is a quantitative (ordinal) value assessed by expert neurologists.
Research sample	Global population, however strongly dominated by people in the United States. Total number of participants was 250. Among them, 137 self-identified as male, and 111 self-identified as female. Majority of the population self-identified them as White, so the dataset is not representative in terms of race. Targeted efforts will be required in the future to collect a representative dataset.
Sampling strategy	No statistical analysis was performed to choose the sample size. Rather, sample size was chosen based on resource availability (i.e., being able to collect ratings from multiple expert neurologists).
Data collection	Data was collected using parktest.net website. In most cases (199 out of 250), participants provided data remotely from their homes without any supervision from the researchers. Some participants (42 out of 250) provided data with clinical supervision.
Timing	Data collection started in 2019 and is still ongoing. We analyzed videos collected from 2019 until 2021. The data collection is done using a website (parktest.net), and anyone can provide data anytime.
Data exclusions	11 videos were excluded from analysis (out of 500 videos) due to extreme quality issues.
Non-participation	Participation involves providing data in one session using the parktest.net website. We do not log events where someone visits the website but does not provide data.
Randomization	Participants were grouped based on their Parkinson's diagnosis status; sex; age; race; and the severity score for the finger-tapping task.

Reporting for specific materials, systems and methods

We require information from authors about some types of materials, experimental systems and methods used in many studies. Here, indicate whether each material, system or method listed is relevant to your study. If you are not sure if a list item applies to your research, read the appropriate section before selecting a response.

Materials & experimental systems

n/a	Involved in the study
☒	☐ Antibodies
☒	☐ Eukaryotic cell lines
☒	☐ Palaeontology and archaeology
☒	☐ Animals and other organisms
☒	☐ Clinical data
☒	☐ Dual use research of concern
☒	☐ Plants

Methods

n/a	Involved in the study
☒	☐ ChIP-seq
☒	☐ Flow cytometry
☒	☐ MRI-based neuroimaging
